# Nano-Cracked Strain Sensor with High Sensitivity and Linearity by Controlling the Crack Arrangement

**DOI:** 10.3390/s19122834

**Published:** 2019-06-25

**Authors:** Hyunsuk Jung, Chan Park, Hyunwoo Lee, Seonguk Hong, Hyonguk Kim, Seong J. Cho

**Affiliations:** School of Mechanical Engineering, Chungnam National University, 99 Daehak-ro, Yuseong-gu, Daejeon 34134, Korea; gustjr33333@naver.com (H.J.); cksdl4608@naver.com (C.P.); goohala9191@naver.com (H.L.); ghdwjdgkr123@naver.com (S.H.); guddnr252@naver.com (H.K.)

**Keywords:** strain sensor, high sensitivity, linearity, pre-strain, sensor-extending process, low hysteresis

## Abstract

Studies on wearable sensors that monitor various movements by attaching them to a body have received considerable attention. Crack-based strain sensors are more sensitive than other sensors. Owing to their high sensitivity, these sensors have been investigated for measuring minute deformations occurring on the skin, such as pulse. However, existing studies have limited sensitivity at low strain range and nonlinearity that renders any calibration process complex and difficult. In this study, we propose a pre-strain and sensor-extending process to improve the sensitivity and linearity of the sensor. By using these pre-strain and sensor-extending processes, we were able to control the morphology and alignment of cracks and regulate the sensitivity and linearity of the sensor. Even if the sensor was fabricated in the same manner, the sensor that involved the pre-strain and extending processes had a sensitivity 100 times greater than normal sensors. Thus, our crack-based strain sensor had high sensitivity (gauge factor > 5000, gauge factor (GF = (△R/R_0_)/ε), linearity, and low hysteresis at low strain (<1% strain). Given its high sensing performance, the sensor can be used to measure micro-deformation, such as pulse wave and voice.

## 1. Introduction

As the aging population is increasing worldwide, interest in healthcare is growing dramatically and many studies are being conducted. Among them are personal health monitoring, motion detection, and soft robots, requiring a wearable strain sensor that is stretchable and skin mountable [1,2,3,4,5]. Using a conventional rigid strain sensor, such as a strain gauge, which is not stretchable and has bad wear sensation, is impractical because, to monitor body deformations, the sensors must be attached to clothes or must be directly attached to the person. For this reason, various types of stretchable strain sensors have been fabricated. Owing to high deformability and conformability on various surfaces of the body, soft silicon elastomers, such as Polydimethylsiloxane (PDMS), Ecoflex [6,7], and thermoplastic resin (e.g., polyurethane (PU) [8,9,10] that is biocompatible, chemically inert, and with different textures) are used as a substrate for sensors. Moreover, metallic (e.g., platinum (PT) [11], gold [12], and silver [13]) and carbon-based nanomaterials [14,15,16] are deposited and transferred to fabricate sensors. To measure the motion of a body precisely, excellent sensitivity and stretchability are necessary. A highly sensitive sensor is needed to measure micro-deformation of a body, such as pulse, voice, and eye flicker, whereas a stretchable sensor is required to measure large deformation of a body, such as bending a finger or knee. Previous studies that measured small body movements have shown low sensitivity [17,18,19,20,21], or high sensitivity but the data obtained exhibited low linearity, limiting the accuracy and reliability of the measurements [22,23,24]. The reason for the data nonlinearity is that the substrate, which has viscoelastic properties, is slowly restored or is permanently deformed. Moreover, interaction between the nanomaterial and polymer make hysteresis [25]. Low hysteresis and linearity are essential for sensors because hysteresis leads to a difficulty in obtaining reliable values during continuous measurement of the sensor. Moreover, complex calibration procedures are essential for linearizing nonlinear sensors. To address these limitations, we propose a novel fabrication processes to improve linearity and sensitivity, and decrease hysteresis, referred to as pre-strain (PS) and sensor-extending (SE) processes. The PS process increases the length of the sensor prior to measurement and then returns to the original length to generate a crack, whereas the SE process extends the sensor to develop a gap between cracks. Regulating the degree of the two methods changes the arrangement of the cracks and characteristics of the sensor. In this study, a simple and inexpensive crack-based strain sensor with high sensitivity and linearity was improved by using the PS and SE methods (GF > 5000, R^2^ > 0.99 at 1% strain). Through comparison with recent studies (Table S1) [13,16,22,24,26,27], our sensor showed excellent sensitivity and linearity. We also confirmed the sensitivity difference among PS, SE, and other existing sensors and confirmed that the sensors can be attached to the radial artery to monitor pulse in real time.

## 2. Materials and Methods

### 2.1. Materials and Fabrication of Sensor

**Figure 1:**
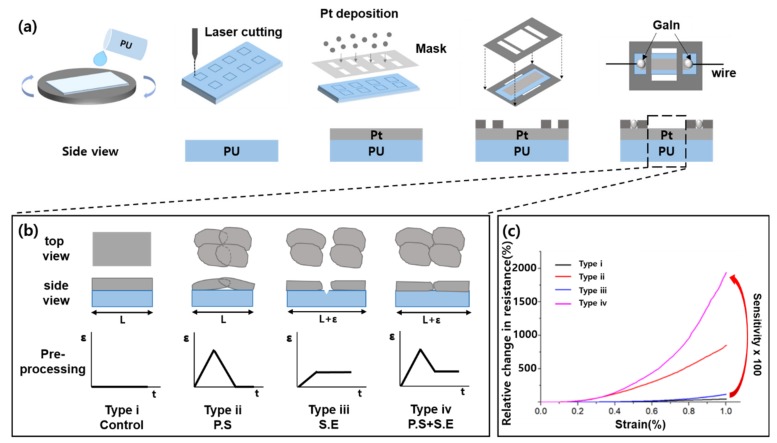
(**a**) Fabrication process of the crack-based strain sensor. (**b**) Schematic of the four sensor types and their pre-processing prior to measurement. (**c**) Relative change in resistance of the four sensor types under 1% strain.

Figure 1a shows the short process of fabricating a crack-based strain sensor. As a sensor substrate, we dissolved a thermoplastic PU (Pallethane 2363-80AE; Lubrizol, Louisville, KY, USA) with 6:4 ratio of tetrahydrofuran (THF) and dimethylformamide (DMF) in a vortex mixer. The dissolved PU solution was applied to the slide glass for a uniform thickness via spin-coating and was cured in an oven at 80 °C for 15 min. To avoid the Pt layer being affected when cutting the PU substrate, we cut the PU membrane prior to the Pt layer deposition. After membrane cutting, a mask was placed on the PU substrate, and the Pt layer was coated by using a conventional sputtering method [8,10]. Then, we placed the PU substrate on the base plate and covered the adhesive top plate to fix the wire. Finally, the wires were placed at both ends of the Pt and connected with gallium–indium (99.99+%, 495425-5G; Sigma Aldrich, Saint Louis, MI, USA) liquid metal or silver epoxy (conductive epoxy, CW2400; Chemtronics, Seongnam, Gyeonggi, Korea) to measure the resistance change of Pt based on the substrate deformation. 

Figure 1b shows four different types of sensors, which are combined PS and SE processes and their electrical characteristics. Type-i is a normal sensor without the PS or SE process, and it has no pre-process, indicating that no crack was on its Pt layer. Type-ii is a sensor after the PS process. In this type, strain was applied, and then it was returned to its original length. It showed overlapping cracks, leading to poor sensitivity at low strain range. Type-iii is a sensor that involves the SE process, which is extended in comparison with the initial sensor length. When the sensor is extended, a gap is observed between the cracks, which can address the crack overlap problem but cause high initial resistance. Type-iv is a sensor that is applied the PS and SE processes sequentially. Cracks were generated but they did not overlap, with only a small gap generated. Figure 1c shows the four types of sensor’s resistance change when 1% strain was applied. Type-iv, which is the hybrid process, showed the highest sensitivity compared to the others.

### 2.2. Evaluation Setup

**Figure 2:**
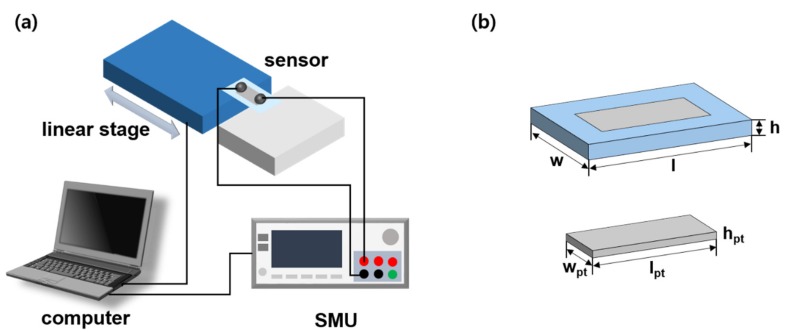
(**a**) Schematic of the sensor-evaluating system. (**b**) Schematic of the sensor.

In measuring and evaluating the sensing performance of the sensors, we developed a sensor-evaluating system. As Figure 2a shows, the wires were connected to both ends of Pt, and then connected to the source measurement unit (SMU) (B2902A; Keysight, Santa Rosa, CA, USA) to measure the resistance change of the membrane sensor. Meanwhile, one side of the sensor was fixed on the jig and the rest is attached on the micro-translation stage (V-528; Physik Instrument, 76228 Karlsruhe, Germany). When the stage moves, deformation occurred in the sensor, and then we measured resistance change based on the deformation. We controlled stage speed, position, and SMU input voltage (1 ~ 2 V, Figure S2) and immediately obtained the resistance change data through the LabVIEW program (NI LabVIEW 2015; National Instruments, Austin, USA). Figure 2b is the schematic of the crack-based sensor, with l = 15 mm, w = 7 mm, h = 20 µm, l_pt_ = 9 mm, w_pt_ = 3 mm, and h_pt_ = 20 nm.

## 3. Results

In this study, we proposed the PS and SE processes to improve sensor sensitivity and linearity. We analyzed the characteristics of the PS and SE processes and assessed the sensing performance of the sensor that is involved with PS, SE, and hybrid processes. The result indicated that the sensor was able to be control the sensitivity and linearity by regulating the hybrid process. By using our novel process, we demonstrated the highly sensitive strain sensor applications.

### 3.1. PS Process and SE Process

**Figure 3:**
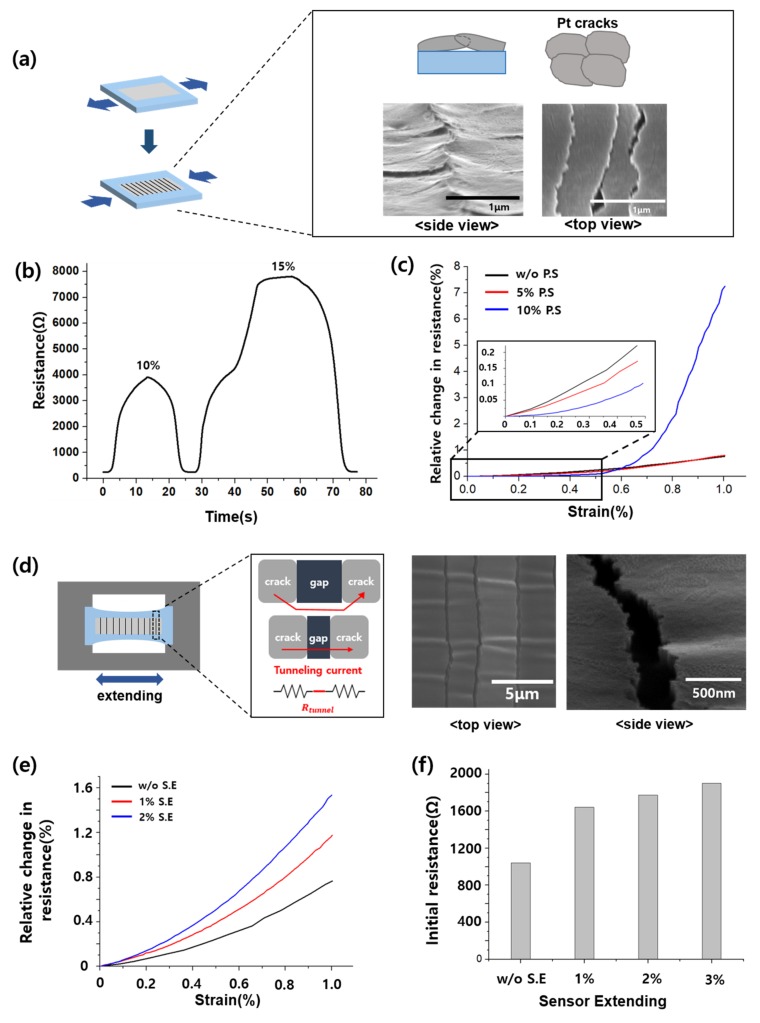
(**a**) Schematic of the pre-strained sensor and its scanning electron microscope (SEM) images. (**b**) Previous strain’s resistance value affects the following strain’s resistance value, (**c**) Shortcoming of pre-strained sensor, (**d**) Schematic of the sensor-extending process, its concept and SEM images of the extended sensor, (**e**) Strain-relative change in resistance graph according to sensor-extending, (**f**) Shortcoming of sensor-extending, high initial resistance.

The PS process is the procedure of generating cracks prior to measurement. As Figure 3a shows, Pt film cracks were generated on the top of PU polymer layers upon stretching. By using the PS process, we were able to improve the reliability and sensitivity of the sensor, because the resistance change in the crack-based sensor was affected by the resistance value at the previous strain. The resistance–strain relationship exhibited history dependence in the form of maximum strain previously reached (Figure 3b). It is expected to result from the Mullins effect. This effect is a particular aspect of the mechanical response of a polymer substrate, indicating that the stress–strain curve changes depend on the maximum load previously generated [28,29]. As Figure 3b shows, when 15% strain is applied, the resistance value at the previous 10% strain is affected while stretching. To address this problem, we proposed the PS process, i.e., applying more than the desired strain value. For example, in the case of sensor with 5% PS process, if the strain is less than 5%, the sensor will not be affected by the previous value. However, the PS process affected the sensitivity of the sensor, as shown in Figure 3c. Figure 3c shows the graph of the relative resistance change rate (= (R–R_0_)/R_0_); up to 1% strain of the sensor did not involve the PS process, whereas 5% and 10% strain of the sensor involved the PS process. All measurements were facilitated using the same sensor. As the value of PS increased, the sensitivity also increased, and the sensor with 10% PS exhibited the best sensitivity. However, the pre-strained sensor was less sensitive than that without the PS sensor under 0.5% strain. We assume that this result is related to the overlapping of the cracks during the PS process. Moreover, the crack overlapped, as observed in the scanning electron microscope (SEM) images of Figure 3a. When the adhesion between the PU substrate and Pt layer is poor, several parts of Pt cracks are detached from the PU substrate while the sensor is stretched. This phenomenon is the reason for the overlapping Pt cracks after the PS process. In the strain range where the cracks overlap, the resistance change rate is small and the sensitivity is low. The larger the PS, the larger the overlap, and the sensitivity is less at the micro-strain.

SE is the process of generating gaps prior to the measurement. Electrons can tunnel through the closely spaced adjacent and overlapping cracks [30], rendering the sensor nonlinear (Figure S4). To prevent the tunneling effect and crack overlap, we slightly stretched the sensor and generated a gap between the cracks. We confirm the gap through the SEM, as shown in Figure 3d. Unlike the PS sensor’s SEM image (Figure 3a), the SE sensor’s cracks did not overlap, but separated from each other. As shown in Figure 3e, the sensor that involves the SE process has higher sensitivity at low strain than the sensor without the SE process. However, the sensors that involved the SE process have small relative change in resistance rate compare with the sensor that involved the PS process. The greater the extension of the sensor, the higher the increase of the initial resistance value, as shown in Figure 3f. Accordingly, the GF and normalized electrical resistance variation value are reduced. In addition, the sensor with the SE process exhibits a low resistance change rate. Therefore, the sensor that involved SE can improve the sensitivity and linearity at low strain compared with that which did not involve the SE sensor, but too much extension may reduce the sensitivity.

### 3.2. Hybrid Process Combined with the PS and SE Processes

**Figure 4:**
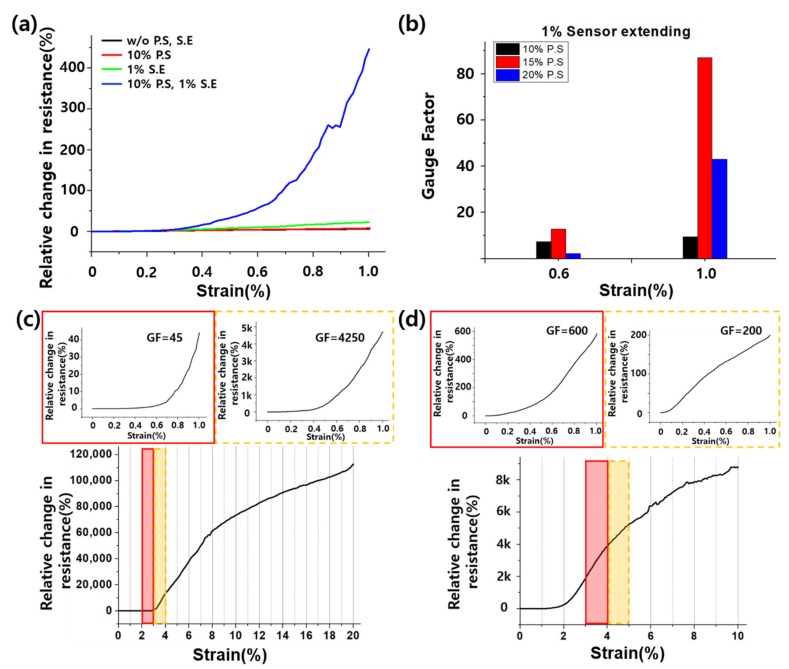
(**a**) Relative resistance change of the four sensor types under 1% strain. (**b**) Gauge factor at 0.6% and 1% strain according to the pre-strain percentage under 1% sensor extension. (**c**) Relative resistance change under 20% strain: red line box, pre-strain 20% and sensor-extending 2% under 1% strain; orange dotted line box, pre-strain 20% and sensor-extending 3% under 1% strain. (**d**) Relative resistance change under 10% strain: red line box, pre-strain 10% and sensor-extending 2% under 1% strain; orange dotted line box, pre-strain 10% and sensor-extending 3% under 1% strain.

We proposed the hybrid process, which is a combination of the two processes, to eliminate disadvantages while retaining advantages. As mentioned previously, we confirmed that the PS and SE processes affect sensor performance. However, the sensor with PS exhibited sensitivity improvement but was nonlinear because of the crack overlap. Meanwhile, the sensor with SE exhibited linearity improvement but has no sensitivity advancement. As Type-iv of Figure 1b shows, SE after PS addresses the crack overlap problem. By using the hybrid process, we can produce a highly sensitive and linear sensor at low strain range. Figure 4a shows the graph of the relative resistance change under 1% strain of the four sensor types. In the graph, the sensor with hybrid process has a remarkable resistance change rate compared to the others. Figure 4b illustrates the graph of the GF values at 0.6% and 1% strains when 1% SE was added after 10%, 15%, and 20% PS, respectively. The sensor with 15% PS had better sensitivity than that with 20% PS. Therefore, the sensitivity was not proportional to PS. An optimal value of PS and SE exists for high sensitivity and linearity. Figure 4c,d indicate that we can regulate the sensitivity. The below graphs of Figure 4c,d show the relative change in resistance when 20% strain and 10% strain was applied each. As mentioned above, PS has two processes—stretching and releasing. The below graphs show the stretching process of PS. By using this graph, we can determine how much SE is necessary for a highly sensitive sensor. The graph in the red lined box shows the resistance change of the 20% PS and 2% SE sensors under 1% strain, whereas the graph in orange dotted-lined box illustrates the resistance change of 20% PS and 3% SE sensor under 1%. The two graphs show a remarkable sensitivity difference. Because the slope of the relative change in the resistance graph denotes the GF, the slope in the red lined box seems much smaller than that in the orange dotted-lined box. As Figure 4d shows, the red lined box has higher sensitivity than the orange dotted-lined box because the red lined box’s slope is higher than that of the orange dotted-lined box. Thus, we were able to assess how much SE should be applied for high sensitivity through the pre-strained sensor’s resistance graph.

### 3.3. Nano-Cracked Strain Sensor with High Sensitivity and Linearity

**Figure 5:**
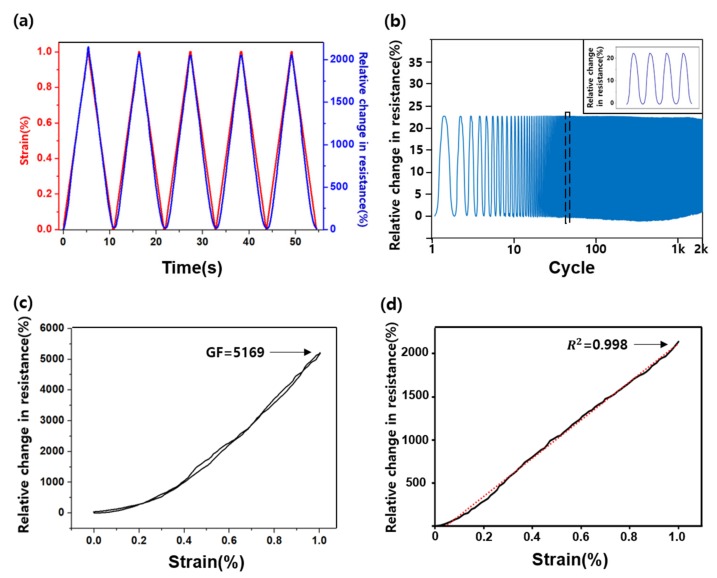
(**a**) Relative resistance change during the cycling at the 0–1% strain. (**b**) Relative resistance change during 2000 cycles (inlet: resistance change at specific cycles). (**c**) High GF strain sensor after the pre-strain and sensor-extending processes. (**d**) Linear strain sensor after the pre-strain and sensor-extending processes.

Figure 5 shows the sensor performance with PS and SE. Figure 5a is the graph showing the relative resistance change rate and 0–1% strain repeated five times based on time. The sensor has fast response time (<25 ms) and recovery time (<30 ms) at 1% strain. The resistance increases and decreases almost along with the deformation of the sensor. However, the peak area under the relative resistance change rate tends to increase sufficiently, caused by the failure to return to the initial state owing to plastic deformation of the PU substrate. Figure 5b is the graph of the change rate of the relative resistance when a 0–0.2% strain is repeated. In addition, obtaining a value similar to the initial value for several thousand repetitive measurements is important when repeating the micro-deformation many times, such as in the case of pulse waves. The cycles were repeated 2000 times, and the resistance change was almost the same. Figure 5b (inlet) shows the enlargement of several cycles. This graph confirmed that the fabricated sensor had a similar value to the initial relative resistance change rate without any significant difference. Figure 5c,d show the high sensitivity and good linearity of the sensors through the changes in PS and SE value. We obtained high sensitivity, with 5169 GF, and low hysteresis, wherein the resistance rate when stretched and relaxed is almost the same. As Figure 5d shows, the sensor has good linearity (R^2^ = 0.998) but has lower sensitivity than that in Figure 5c. Previous studies demonstrated poor linearity when the sensor has high sensitivity, and poor sensitivity when the sensor has good linearity (Table S1). Unlike the results of these studies, our sensor has high sensitivity and good linearity at once. 

**Figure 6:**
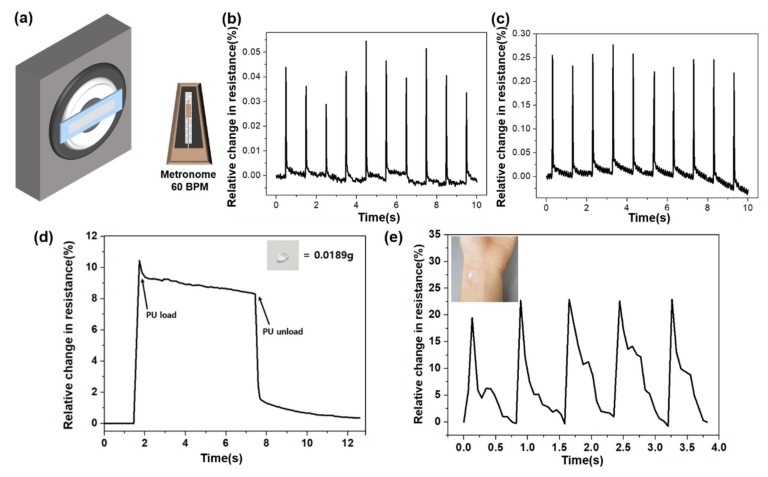
(**a**) Schematic of the sensor attached on a speaker, generating 60 BPM metronome. (**b**–**c**) Difference in the relative resistance change between with and without the hybrid process. (**d**) Relative resistance change when PU bead loading and unloading. (**e**) Relative resistance change of the pulse signal (inlet: photograph of the attached spot).

The fabricated sensor sensitively detected micro-deformations. To demonstrate this, we attached the sensor to a speaker, and then measured the resistance change, as shown in Figure 6a. A 60 BPM metronome sound was outputted from the speaker. As the volume of the speaker increased, the deformation of the sensor increased. Thus, the peak relative change in resistance value of Figure S6b was smaller than the peak value in Figure S6d. The deformation of the sensor caused by volume difference was almost the same, but the sensor can detect the volume difference. The reason for the resistance decrease with time is the adhesive force between Pt and PU layers was altered by repeated measurement. Figure 6b,c show the difference in the relative resistance change between the normal and hybrid sensor. As mentioned previously, the sensor that involved the hybrid process is more sensitive than the sensor without the hybrid process. Thus, the fabricated sensor has the possibility to measure a pulse that causes a very small deformation to the skin. However, in the case of pulse, unlike the previous strain measurement, the sensor deformation occurred above the attachment part of the sensor. Prior to measuring the pulse, we confirmed that the sensor can sensitively measure micro-deformations from bottom to top. As Figure 6d shows, the relative resistance change of the sensor was measured when one ultra-lightweight PU bead of 0.0189 g was placed on the sensor. After confirming that the fabricated sensor could measure micro-deformation, we measured the pulse signal. Figure 6e shows the pulse measurement by attaching the sensor on the wrist. The output signal exhibited a matching waveform of percussion, diastolic, and tidal wave of a human pulse.

## 4. Conclusions

In this study, we used the PS and SE processes to address the crack-based sensor problem, such as low sensitivity at low strain, nonlinearity, and hysteresis. We confirmed that the sensor has superior performance than the existing sensor and has good sensor performance regarding high sensitivity (GF > 5000 at <1% strain), linearity (R^2^ > 0.99), and repeatability (>2000 cycles), and low hysteresis at low strain. This result confirms that small, repetitive deformations, such as pulse, can be measured with high sensitivity. Because the pulse waveform is non-invasive and can be used as an index to predict cardiovascular diseases, the sensitivity and accuracy of the sensor are important. Thus, the sensor produced is expected to be more practical, sensitive, and reliable than the existing ones.

## Supplementary Materials

The following are available online at https://www.mdpi.com/1424-8220/19/12/2834/s1, Figure S1: Experimental setup for strain sensor evaluation, Figure S2. Initial resistance-voltage (R0-V) graph of crack-based strain sensor in 0 % strain. Figure S3: Gauge factor difference between sensor with SE and without SE after 5% PS, Figure S4: Current–Voltage (I–V) curve of crack-based sensor, Figure S5: Cycle tests, Figure S6: Metronome tests, Figure S6: Mullins effect, Table S1: Comparison of sensing performance with other literature.
