# Supplementary material for: Nano-Cracked Strain Sensor with High Sensitivity and Linearity by Controlling the Crack Arrangement
Source: Sensors (Basel). 2019 Jun 25;19(12):2834. doi: 10.3390/s19122834 (PMC6631595; doi:10.3390/s19122834)
Supplement: Supplementary file 1 [file sensors-19-02834-s001.pdf]

sensors
MDPI

Supplementary information

Nano-Cracked Strain Sensor with High Sensitivity and Linearity by Controlling the Crack Arrangement

Hyunsuk Jung †, Chan Park †, Hyunwoo Lee, Seonguk Hong, Hyonguk Kim and Seong J. Cho *

School of Mechanical Engineering, Chungnam National University, 99 Daehak-ro, Yuseong-gu, Daejeon 34134, Korea; gustjr33333@naver.com (H.J.); cksdl4608@naver.com (C.P.); goohala9191@naver.com (H.L.); ghdwjdgkr123@naver.com (S.H.); guddnr252@naver.com (H.K.)
† These authors contributed equally to this work.
* Correspondence: scho@cnu.ac.kr; Tel.: +82-42-821-5648

Received: 27 May 2019; Accepted: 24 June 2019; Published: date

Table S1. Comparison of sensing performance with other literatures.

Reference	Sensing materials / Substrate	Gauge Factor (<1% strain)	Linearity at 1% strain
**1**	Pt /PUA	<800	Nonlinear
**2**	CBs / Ecoflex	<0.1	Linear
**3**	Graphite / Ecoflex	26.9	Linear
**4**	AgNWs / PDMS ZnONWs	<0.1	Linear
**5**	CNTs-PEDOT:PSS / PU	<10	Linear
**6**	CBs / PDMS	<30	Linear
**This work**	Pt / PU	>5,000	Linear ($R^2 > 0.99$)

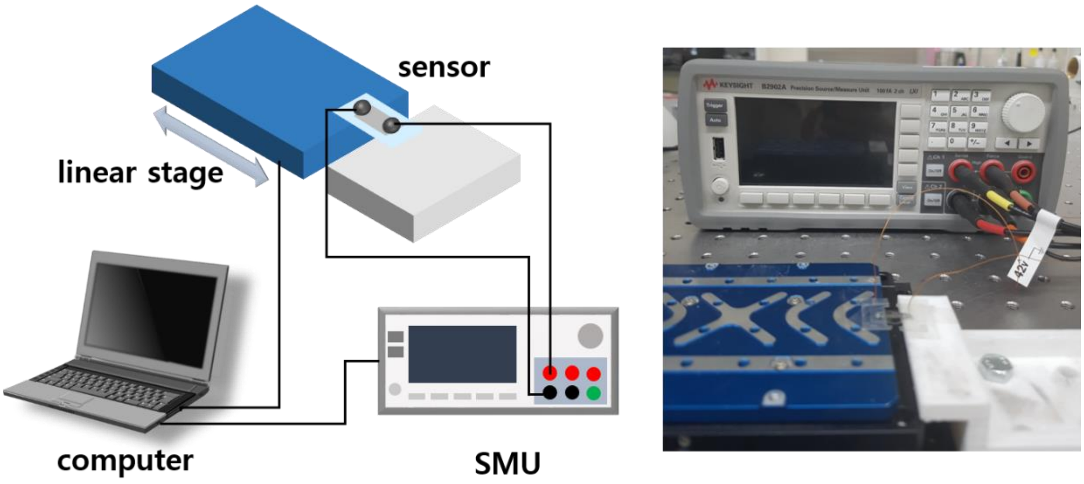

Figure S1. Experimental setup for strain sensor evaluation.

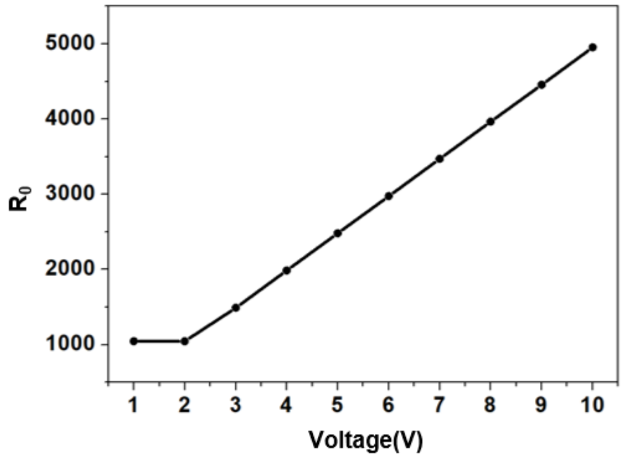

Figure S2. Initial resistance-voltage (R0-V) graph of crack-based strain sensor in 0 % strain.

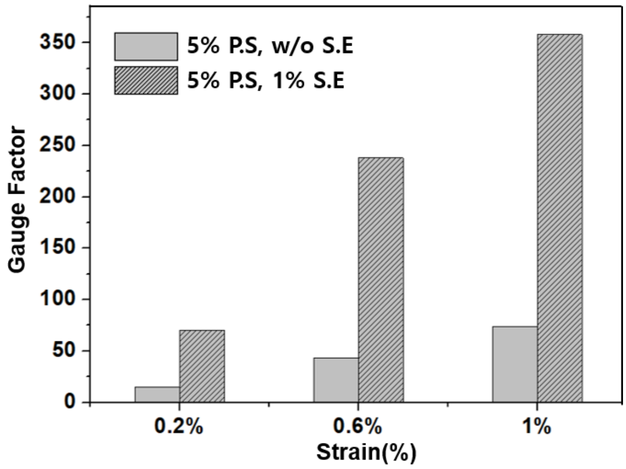

Figure S3. Gauge Factor difference between sensor with S.E. and without S.E. after 5% P.S.

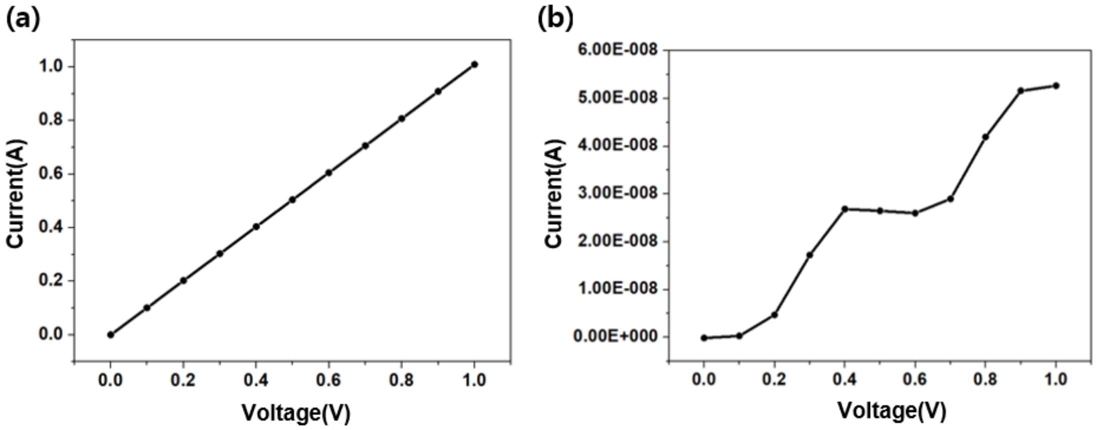

Figure S4. Current-Voltage (I-V) curve of crack-based sensor (**a**) current-voltage (I-V) curve of crack-based strain sensor at 0 % strain. (**b**) current-voltage (I-V) curve of crack-based strain sensor at 1 % strain.

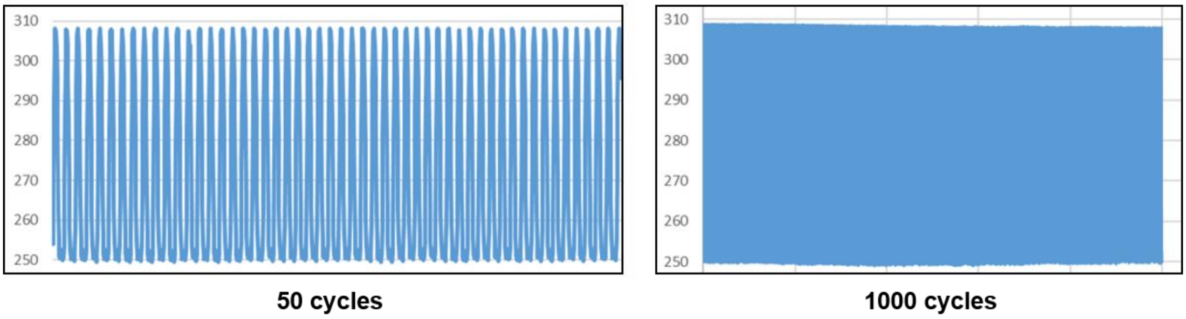

Figure S5. Cycle tests.

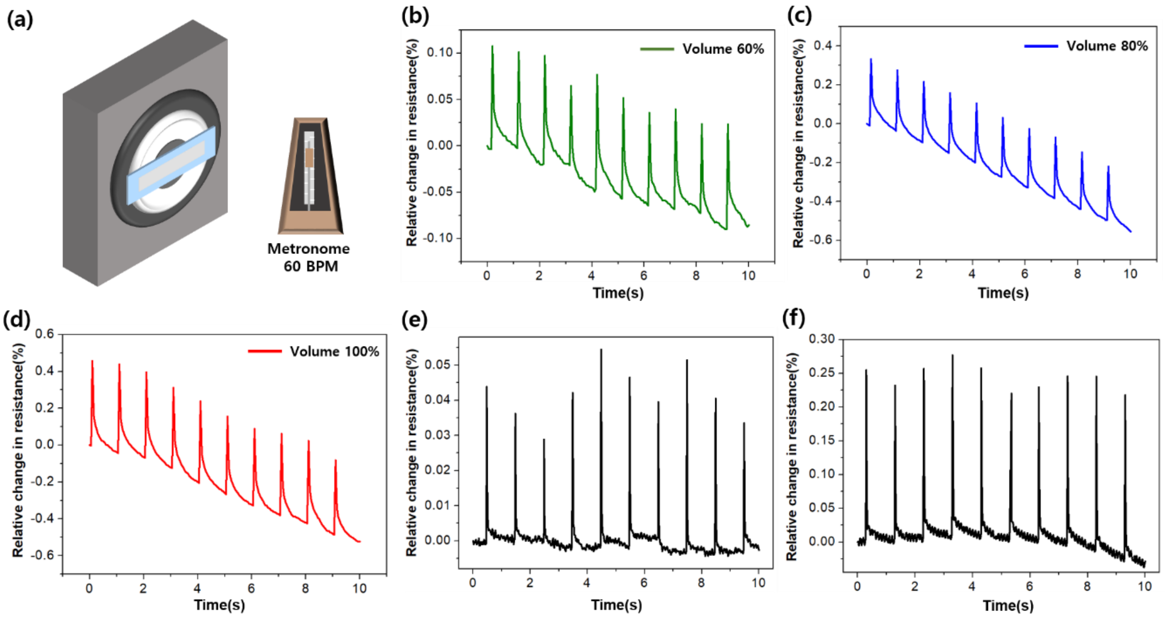

Figure S6. (**a**) Schematic of sensor attached on a speaker which generate 60 bpm metronome, (**b**-**d**) Relative change in resistance according to speaker volume, (**e**-f) Relative change in resistance when didn't do P.S, S.E and did P.S, S.E.

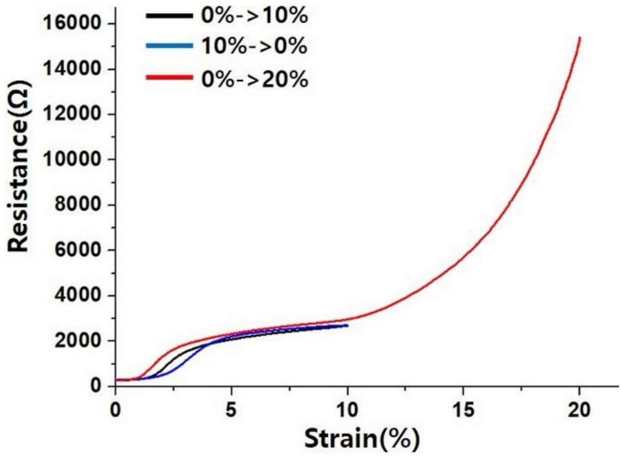

Figure S7. Mullins effect.

References

1. Park, B.; Kim, J.; Kang, D.; Jeong, C.; Kim, K.S.; Kim, J.U.; Yoo, P.J.; Kim, T. Dramatically Enhanced Mechanosensitivity and Signal-to-Noise Ratio of Nanoscale Crack-Based Sensors: Effect of Crack Depth. *Adv. Mater.* **2016**, 28, 8130–8137.
2. Wang, Z.; Huang, Y.; Sun, J.; Huang, Y.; Hu, H.; Jiang, R.; Gai, W.; Li, G.; Zhi, C. Polyurethane/Cotton/Carbon Nanotubes Core-Spun Yarn as High Reliability Stretchable Strain Sensor for Human Motion Detection. *ACS Appl. Mater. Interfaces* **2016**, 8, 24837–24843.
3. Amjadi, M.; Turan, M.; Clementson, C.P.; Sitti, M. Parallel Microcracks-based Ultrasensitive and Highly Stretchable Strain Sensors. *ACS Appl. Mater. Interfaces* **2016**, 8, 5618–5626.
4. Amjadi, M.; Pichitpajongkit, A.; Lee, S.; Ryu, S.; Park, I. Highly stretchable and sensitive strain sensor based on silver nanowire-elastomer nanocomposite. *ACS Nano* **2014**, 8, 5154–5163.
5. Roh, E.; Hwang, B.U.; Kim, D.; Kim, B.Y.; Lee, N.E. Stretchable, Transparent, Ultrasensitive, and Patchable Strain Sensor for Human–Machine Interfaces Comprising a Nanohybrid of Carbon Nanotubes and Conductive Elastomers. *ACS Nano* **2015**, 9, 6252–6261.
6. Lu, N.; Lu, C.; Yang, S.; Rogers, J.A. Highly Sensitive Skin-Mountable Strain Gauges Based Entirely on Elastomers. *Adv. Funct. Mater.* **2012**, 22, 4044–4050.
